# The UK Midwifery Study System (UKMidSS): a programme of work to establish a research infrastructure to carry out national studies of uncommon conditions and events in midwifery units

**DOI:** 10.1186/s12884-016-0868-1

**Published:** 2016-04-14

**Authors:** Rachel E Rowe, Jennifer J Kurinczuk, Jennifer Hollowell, Marian Knight

**Affiliations:** National Perinatal Epidemiology Unit, Nuffield Department of Population Health, University of Oxford, Old Road Campus, Oxford, OX3 7LF UK

**Keywords:** Midwifery, Birth centre, Obesity, Neonatal unit admission

## Abstract

**Background:**

Midwifery-led care during labour and birth in the UK is increasingly important given national commitments to choice of place of birth, reduction of unnecessary intervention and improving women’s experience of care, and evidence on safety and benefits for ‘low risk’ women. Further evidence is needed on safety and potential benefits of midwifery-led care for some groups of ‘higher risk’ women and about uncommon adverse outcomes or ‘near-miss’ events. Uncommon obstetric events and conditions have been investigated since 2005 using the UK Obstetric Surveillance System. This programme of research will establish the UK Midwifery Study System (UKMidSS) in all UK alongside midwifery units (AMUs) and carry out the first two UKMidSS studies investigating: (i) outcomes in severely obese women admitted to AMUs, and (ii) risk factors for neonatal unit admission following birth in an AMU.

**Methods:**

We will carry out national cohort and case-control studies using UKMidSS, a national data collection platform which we will establish to collect anonymised information from all UK AMUs. Reporting midwives in each AMU will actively report cases or nil returns in response to monthly notification emails. Denominator data on the number of women admitted to and giving birth in each AMU will also be collected.

Anonymised data on risk factors, management and outcomes for cases and controls/comparators as appropriate for each study, will be collected electronically using information from medical records.

We will calculate incidence and prevalence with 95 % confidence intervals (CIs), tabulate descriptive data using frequencies and proportions, and use logistic regression to estimate odds ratios with 95 % CIs comparing specific outcomes in case and comparison women and to investigate risk factors for conditions or outcomes.

**Discussion:**

As the first national infrastructure facilitating research into uncommon events and conditions in women starting labour in midwifery-led settings, UKMidSS builds on the success of other national research systems. UKMidSS studies will extend the evidence base regarding the quality and safety of midwifery-led intrapartum care and investigate extending the benefits of midwifery-led care to more women. As a national collaboration of midwives contributing to high quality research, UKMidSS will provide an infrastructure to support midwifery research capacity development.

## Background

The English Department of Health’s maternity care strategy includes commitments to choice of place of birth, the reduction of interventions and improving women’s experience of care [1–4]. In this context midwifery-led care during labour and birth is increasingly important. In healthy women with straight forward pregnancies (‘low risk’ women) planned birth in a midwifery-led setting is associated with a reduced risk of labour and birth interventions, including augmentation, epidural/spinal analgesia, general anaesthesia and instrumental or operative delivery [5–7]. Outcomes for babies of ‘low risk’ women planning birth in midwifery units are comparable with those for babies of women planning obstetric unit (OU) birth [5, 6]. Women planning birth in midwifery-led settings also report higher levels of satisfaction with their care [7].

In response to the policy imperatives on choice and women’s experience, and the evidence on safety, the number of midwifery units in England increased from 87 in 2007 [8] to 152 in 2013, with most of that increase in alongside midwifery units (AMUs) located on the same site as an OU [9]. In 2012 available data suggested that around 15 % of pregnant women in England planned to give birth in a midwifery-led setting with around 80 % of these planning birth in an AMU [5, 9]. Between half and two thirds of pregnant women are estimated to be at ‘low risk’ of complications during labour and birth and therefore are eligible for midwifery-led care, indicating significant potential for further increases [10, 11]. Current national guidelines recommend that women may choose any birth setting, but that for ‘low risk’ women midwifery units are “particularly suitable” [12].

Against a background of evidence about the benefits of midwifery-led care for ‘low risk’ women and increasing demand for and provision of midwifery units in the UK, there is a need to extend the evidence base regarding the quality and safety of midwifery-led intrapartum care and to investigate the risks and benefits of extending midwifery-led care to more women. Routine data sources have not historically enabled accurate identification of births in AMUs and are not sufficiently detailed or comprehensive to investigate risk factors for uncommon conditions or events, to control for potential confounders or to investigate management and any association with outcome. High quality evidence to inform clinical practice is therefore lacking. Studies based in individual units require retrospective review of many years of data, may be compromised by changes in practice over time and may not be generalisable to other units because of differences in the socio-demographics of the populations served and in clinical practice.

In obstetrics, uncommon events, disorders and outcomes have been investigated since 2005 in national studies carried out through the UK Obstetric Surveillance System (UKOSS), led from the National Perinatal Epidemiology Unit (NPEU) at the University of Oxford. [13] UKOSS covers all consultant-led OUs in the UK and allows for identification and study of cases of uncommon events and disorders, including ‘near-miss’ events, and appropriate comparison women. Studies carried out using UKOSS have provided robust, promptly reported evidence on incidence, risk factors, clinical practice, management and outcome for uncommon conditions, events and outcomes reported in over 40 peer reviewed publications and annual reports [14].

This protocol describes a programme of work to investigate uncommon events and conditions in AMUs through the development and establishment of the UK Midwifery Study System (UKMidSS), a national data collection platform for all AMUs in the UK, using methods similar to those used for UKOSS. UKMidSS will focus on AMUs because that is where the majority of births in midwifery-led settings are planned and take place, but there may be the opportunity, with additional resources, to expand to include other midwifery-led settings (freestanding midwifery units and planned home births) in the future.

The research infrastructure established by UKMidSS will facilitate a rolling programme of national cohort and case control studies. The first two studies to be carried out using UKMidSS are described in this protocol; future studies will follow the same methods and approach. The research questions which can be addressed by UKMidSS studies include, but are not limited to: (i) investigating outcomes in groups of women who currently may be regarded as at higher risk of complications but who nevertheless plan birth in an AMU, and (ii) investigating incidence and quantifying risk factors for adverse outcomes or ‘near-miss’ events.

The first UKMidSS study will investigate outcomes in severely obese women, i.e. with a body mass index (BMI) >35 kg/m^2^ starting labour care in an AMU. Maternal obesity is recognised as a risk factor for complications and adverse outcomes of pregnancy, labour and birth [15, 16] and, as a consequence, UK national clinical guidelines advise that severely obese women should plan birth in an OU to reduce these risks [12]. Recent research on women planning birth in OUs indicates that ‘otherwise healthy’ obese multiparous women may have lower intrapartum-related risks than was previously thought [17], and suggests that some severely obese women may safely be managed in an AMU. The estimated prevalence of severe obesity in women planning AMU birth was 1 % in 2008–10 [5], but anecdotal evidence from the Royal College of Midwives and from NHS hospitals suggests that increasing numbers of severely obese women are planning birth in midwifery units. We do not know how many severely obese women currently plan birth in an AMU in the UK and there is no evidence on management of their labour, labour complications or maternal and neonatal outcomes on which to base clinical guidelines and AMU admission criteria or to inform clinical practice and women’s decision-making.

The second UKMidSS study will investigate women who have a baby admitted to a neonatal unit following birth in an AMU. Admission of full-term babies to neonatal care is a key indicator of the safety of maternity care [4]. National clinical guidance on intrapartum care recommends transfer to an obstetric unit when certain complications occur during labour in a midwifery-led setting [12]. In a national cohort study of the safety of different settings for birth in England, around 40 % of adverse perinatal outcomes in births planned in midwifery-led settings occurred in births which took place in the original setting, that is where no transfer took place [5]. Neonatal admission following birth in an AMU is therefore a potential indicator of a ‘near-miss’ event where different management might have made a difference to outcome. We do not know how many women have babies in an AMU who are admitted to a neonatal unit or the extent to which management may be associated with outcome in these cases. While uncommon, events such as this can have a significant effect on women, families and staff; investigation of management and risk factors associated with these events has the potential to inform improvements in the safety of midwifery-led care.

## Aim and objectives

The aim of this programme of research is to generate evidence about care and outcomes in AMUs to strengthen the development of safe, high quality midwifery-led care and improve outcomes for mothers and babies.

In order to achieve this aim, we will develop and establish a national reporting and research system involving all AMUs in the UK (UKMidSS) and conduct a series of national studies using this system.

## Methods

National cohort and case-control studies will be carried out using a national reporting and research system (UKMidSS) which will be established to collect anonymised information from all AMUs in the UK. The key components of UKMidSS will be:

- A network of reporting midwives (one or two in each AMU in the UK)
- Active monthly notification of cases for UKMidSS studies
- Monthly reporting of denominator data
- A rolling programme of observational studies
- Collection of anonymised data about cases and controls or comparison women

### Case identification

A monthly email will be sent to UKMidSS reporting midwives who will be recruited in all AMUs in the UK. This email will indicate the exposure condition or event for each study currently being carried out using UKMidSS and ask the midwife to respond indicating the number of cases meeting the case eligibility criteria that have occurred in their unit during the previous calendar month, or report a nil return if they have no cases to report. In this way, non-responding AMUs will be identified and follow up reminders will be sent. Response rates will be monitored month by month.

### Reporting of denominator data

Historically, routine data systems have not reliably recorded planned place of birth at the start of care in labour, nor do they enable accurate identification of the number of births taking place in AMUs because AMU data are often combined in routine systems with data from the associated OU. For these reasons reporting midwives will also be asked to report the number of women admitted for labour care and giving birth in the AMU. This will enable estimation of prevalence/incidence for each study.

### Data collection

UKMidSS will facilitate a programme of national observational studies to investigate incidence, prevalence and outcomes of uncommon conditions and events in midwifery units. Initial data collection will focus on two studies, summarised here. Future studies will use the same methods and procedures.

Data collection for the first two studies will focus on the following:

#### Study 1: Severely obese women starting labour care in an AMU: prevalence, management and outcomes

This national cohort study will estimate the number and proportion of women starting labour care in AMUs who have a BMI > 35 kg/m^2^, and compare their socio-demographic and clinical characteristics, progress of labour and maternal and neonatal outcomes with women of normal weight starting labour care in AMUs. Women identified as being severely obese (with a BMI > 35 kg/m^2^) at booking and admitted for labour care in the AMU, and a comparison cohort of women of normal weight planning birth in the same AMU, will be identified using UKMidSS over a period of one year, using the procedure described above. Comparison women will be selected based on methods similar to those used in UKOSS studies, e.g. defined as the two women of normal weight starting labour care in the unit immediately before the obese woman in the same AMU. Data on maternal socio-demographic and clinical characteristics, progress and management of labour, any complications and neonatal and maternal outcomes will be collected on a study specific web-based data collection form.

#### Study 2: Women whose babies are admitted to a neonatal unit following birth in an AMU: incidence, management, risk factors and outcomes

This national population-based case-control study will estimate the national incidence of neonatal admission following birth in an AMU, describe management of these cases in relation to national guidelines for intrapartum care, identify risk factors associated with admission and identify factors associated with adverse outcome in babies admitted to neonatal care. Women whose baby was admitted to neonatal care following birth occurring in an AMU will be identified using UKMidSS over a period of one year, as described above. Controls will be defined as the two births in the same AMU immediately preceding the case, where the baby was not admitted to neonatal care. Data on maternal socio-demographic and clinical characteristics, progress and management of labour, any complications, reasons for admission and neonatal and maternal outcomes will be collected on cases and controls using a study specific web-based data collection form.

### Data collection process

The data collection process for all studies is as follows. On receiving notification of a case, the central UKMidSS data management system at the NPEU will allocate a unique number to that case and send the reporting midwife a link to a secure web-based data collection form. Study-specific data collection forms will be developed for each study, based on a standard template. Data collection forms will enable confirmation of the appropriate case definition, collect information on risk factors, management and outcomes as appropriate for each study, and will be completed using information from medical records only. Only anonymised data will be collected; no names, addresses, postcodes, dates of birth, hospital or NHS numbers will be sought. The reporting midwife in each AMU will be asked to keep a record of each case number linking it to identifying information in order to avoid duplication of reporting. If data are not entered on the data collection system within six weeks of case notification a reminder email will be sent. If there is no response after a further four weeks, the midwife will be contacted by telephone.

Controls or comparison women (where required) will be identified from the same AMUs, as appropriate according to the design of the specific study. Anonymised data on controls will be collected as described above for cases.

### Statistics and analysis

#### Statistical methods

Incidence and prevalence will be calculated with 95 % confidence intervals (CIs), including for example, the prevalence of obesity in women starting labour care in AMUs and the incidence of neonatal admission following birth in an AMU. Socio-demographic and clinical characteristics and secondary outcomes will be tabulated, presenting frequencies and proportions with 95 % CIs. These will include, for example: management and intervention during labour; transfer and timing of decision-making around transfer; mode of birth; adverse maternal outcomes, including blood transfusion or admission to higher level care; adverse neonatal outcomes, including neonatal unit admission. Logistic regression will be used to estimate odds ratios (ORs) with 95 % CIs comparing specific outcomes in case and comparison women, to investigate risk factors for conditions or outcomes, adjusting as appropriate for potential confounders such as maternal age, parity, socio-economic status, ethnicity and smoking status.

#### Sample size and power

Study sizes will be determined by the annual population of women planning birth or giving birth in AMUs in the UK, estimated at present to be around 70,000.

For the obesity study, evidence from the Birthplace cohort study in 2008-10 indicated a prevalence of severe obesity (BMI < 35 kg/m^2^) in women planning birth in an AMU of just less than 1 % [5]. Based on an estimated 70,000 planned births in AMUs in the UK every year, this would result in an estimated 690 annual cases (prevalence 1 %, 95 % CI 0.09–1.01 %). Study durations are planned to provide sufficient ‘cases’ and controls to give studies at least 80 % power at the 5 % level of statistical significance for a range of analyses. For example, using the study primary outcome (a measure used elsewhere which captures intrapartum interventions and adverse maternal outcomes requiring obstetric care) [17] and assuming these sample sizes and a 21 % event rate in the comparison (normal weight) group [17], this study will have 80 % power at the 5 % level to detect an OR of 1.4 or greater associated with the primary outcome, between the obese and normal weight groups. For a less common outcome, such as neonatal unit admission, assuming that 2 % of planned AMU births to normal weight women result in an admission to the neonatal unit [5], this study will have 80 % power at the 5 % level to detect an OR of 2.4 or greater for neonatal unit admission between the obese and normal weight groups.

For the neonatal unit admission study, exploratory analysis of the Birthplace cohort study data indicated that there were 203 neonatal admissions following birth in an AMU, which gives an estimated incidence of 1.5 % and an estimated 1050 cases in this study. Table 1 illustrates the ORs detectable by this study for different frequencies of a particular exposure variable with 80 % power at the 5 % level of statistical significance.

**Table 1:** Odds ratios detectable for given exposure variable frequencies^1^

Exposure variable frequency in control group	Odds ratio detectable by the study
5 %	≥1.7
10 %	≥1.5
15 %	≥1.4
20 %	≥1.3
30 %	≥1.3
50 %	≥1.3
80 %	≥1.4

^1^ 80 % power at p < 0.05

### Research management and governance

The UKMidSS Steering Group will have responsibility for overall strategic direction and governance of the programme and will meet face-to-face annually with four-monthly teleconferences. This group will be made up of consultant/senior midwives from the constituent countries of the UK, an obstetrician, a neonatologist, representatives of the Royal College of Midwives and the Royal College of Obstetricians and Gynaecologists, lay members and the UKMidSS Chief Investigator and Co-investigators. Protocols for any future studies to be carried out using UKMidSS, will be developed in consultation with the UKMidSS Steering Group, which will oversee their design and conduct and, in future, also consider applications from clinicians and other researchers to carry out other suitable studies using the system.

### Patient and public involvement

Representatives of maternity services user groups were consulted in the development of the funding application for this programme of work. A Service User Advisory Group for UKMidSS will meet annually with interim teleconferences/email consultation in between as required. Two members of this group will also sit on the Steering Group.

### Ethical considerations and approval

The principal ethical consideration raised by this programme of work is around consent. As described above, these are observational studies and data collection will be limited to information from medical records only, will not involve the active participation of women and will not include any identifying information (names, addresses, postcodes, dates of birth, NHS or hospital numbers). The responsibility for care of women included in studies carried out using UKMidSS will remain with their usual clinical team. Data will be collected from the clinical team after the event or outcome of interest has occurred; all women will receive standard care.

Furthermore, the generalisability of the studies to be carried out relies on being able to calculate accurate and unbiased estimates of incidence, management practices and outcomes. For this reason it is essential that data are collected on all cases occurring in the population; the process of seeking individual consent from women in these circumstances would mean that this would not be possible and would be likely to introduce substantial bias to frequency and effect size estimates. The Confidentiality Advisory Group (CAG) of the NHS Health Research Authority (HRA) (formerly the National Information Governance Board and the Patient Information Advisory Group) considers that organisations seeking to use patient information for research purposes may collect anonymised or pseudonymised data without consent [18]. Collecting anonymised data as proposed in the absence of consent is unlikely to cause significant harm for the individuals whose data are included in UKMidSS studies.

This programme of work complies with the Helsinki Declaration and was reviewed and approved by the National Research Ethics Service Committee South West-Frenchay (REC ref. 15/SW/0166) in May 2015. This protocol is based on the REC-approved protocol, edited to conform to journal requirements.

## Discussion

Building on the success of other national surveillance systems including the British Paediatric Surveillance Unit [19] and UKOSS [20] in the UK and similar systems in other countries [21], we believe UKMidSS will be the first national infrastructure facilitating research into uncommon events and conditions in women planning birth in midwifery-led settings. With the growing importance of midwifery-led settings in maternity care UKMidSS represents an opportunity to extend the evidence base regarding the quality and safety of midwifery-led intrapartum care and to investigate the risks and benefits of midwifery-led care for different groups of women. By establishing a national collaboration of reporting midwives across the UK and providing robust, promptly reported evidence on questions of relevance to care in midwifery-led settings, UKMidSS will raise the profile of research in midwifery-led settings. As UKMidSS develops, the opportunity for midwives and maternity care researchers to apply to carry out studies using the UKMidSS infrastructure will help support midwifery research capacity development and enable further high quality research.

## Abbreviations

AMU: alongside midwifery unit
BMI: body mass index
CI: confidence interval
OU: obstetric unit
REC: Research Ethics Committee
UKMidSS: UK Midwifery Study System
UKOSS: UK Obstetric Surveillance System
